# Transarterial Embolization for Chronic Postsurgical or Posttraumatic Pain of Musculoskeletal Origin: Clinical Outcomes and Imaging Correlates

**DOI:** 10.3390/life15081208

**Published:** 2025-07-29

**Authors:** Zi-Rui Huang, Pei-Yi Chen, Neng-Yu Chiu, Sheng-Chieh Lin, Bow Wang, Jui-An Lin, Keng-Wei Liang

**Affiliations:** 1 Department of Physical Medicine and Rehabilitation, Chung Shan Medical University Hospital, Taichung 402306, Taiwan; proa54041089@gmail.com; 2 Department of Physical Medicine and Rehabilitation, Kinmen Hospital, Ministry of Health and Welfare, Kinmen 89142, Taiwan; miaggiochen@gmail.com; 3 Department of Medical Imaging, Chung Shan Medical University Hospital, Taichung 402306, Taiwan; noelchiu2009@gmail.com; 4 Department of Orthopedics, Chung Shan Medical University Hospital, Taichung 402306, Taiwan; phoenix33343@gmail.com; 5 Institute of Medicine, Chung Shan Medical University, Taichung 402306, Taiwan; 6 Department of Medical Imaging, National Cheng Kung University Hospital, Tainan 701401, Taiwan; wangbow1227@gmail.com; 7 College of Medicine, National Cheng Kung University, Tainan 701401, Taiwan; 8 Department of Anesthesiology, Chung Shan Medical University Hospital, Taichung 402306, Taiwan; 9 Department of Anesthesiology, School of Medicine, Chung Shan Medical University, Taichung 402306, Taiwan; 10 Center for Regional Anesthesia and Pain Management, Chung Shan Medical University, Taichung 402306, Taiwan; 11 Department of Anesthesiology, School of Medicine, National Defense Medical Center, Taipei 114201, Taiwan; 12 Department of Anesthesiology, School of Medicine, College of Medicine, Taipei Medical University, Taipei 11031, Taiwan; 13 School of Medicine, Chung Shan Medical University, Taichung 402306, Taiwan

**Keywords:** angiogenesis, embolization, postoperative chronic pain, musculoskeletal disorders, magnetic resonance imaging, minimally invasive procedure

## Abstract

Chronic postsurgical or posttraumatic pain (CPSP) is a persistent pain condition lasting beyond three months after tissue injury, often associated with neuropathic features and pathological angiogenesis. This study investigated the feasibility, safety, and therapeutic potential of transarterial embolization (TAE) in patients with CPSP arising from prior musculoskeletal surgeries or interventions. Six patients with refractory pain and imaging evidence of abnormal neovascularization were retrospectively reviewed. TAE was performed using imipenem/cilastatin particles to selectively target pathological vasculature. Eleven procedures were conducted, achieving 100% technical and clinical success. Mean Numeric Rating Scale scores improved significantly from 7.8 at baseline to 1.3 at final follow-up (*p* < 0.001). No major adverse events occurred, and follow-up imaging demonstrated resolution of inflammation in selected cases. These results support the role of TAE as a minimally invasive treatment option for intervention-related CPSP involving the musculoskeletal system, and further prospective studies are warranted.

## 1. Introduction

Chronic postsurgical pain (CPSP) is defined as pain that arises or worsens following surgery or tissue injury and persists beyond the expected healing period, typically for at least three months [1]. It is distinct from acute postoperative pain or pre-existing conditions. Reported incidence rates of CPSP vary widely—from 5% to 85%—because of inconsistent definitions and methodological heterogeneity [2]. CPSP is a complex and multifactorial condition involving peripheral and central sensitization as well as inflammatory and immune-mediated changes in neuronal and soft tissue. Many patients exhibit neuropathic features such as hyperalgesia and allodynia [3].

The management of CPSP remains challenging, often requiring a multidisciplinary approach including pharmacologic therapy, physical rehabilitation, and cognitive behavioral interventions. Although regional anesthesia and spinal cord stimulation have shown efficacy, other interventional procedures have yet to be firmly established in this setting [3]. Transarterial embolization (TAE) has emerged as a promising modality for managing chronic musculoskeletal pain by targeting pathological angiogenesis. Its analgesic and anti-inflammatory effects have been demonstrated in both preclinical studies and clinical trials, particularly in conditions such as knee osteoarthritis and adhesive capsulitis [4,5,6,7,8]. More recently, TAE has been used to treat refractory postarthroplasty pain and secondary frozen shoulder [9,10] and is gaining interest as part of multimodal strategies for treatment-refractory pain [11].

Based on our clinical experience, a subset of patients with CPSP presents with imaging evidence of ongoing inflammation and abnormal neovascularization, often visualized on magnetic resonance imaging (MRI) as high T2 signal intensity or increased contrast enhancement. These findings suggest a potential therapeutic role for TAE in this population. However, its effectiveness in CPSP has not been formally evaluated. This study aims to assess the feasibility, safety, and clinical outcomes of TAE in patients with CPSP associated with pathological angiogenesis after musculoskeletal intervention. Additionally, we explore the integration of TAE into a multimodal treatment framework for refractory CPSP cases.

## 2. Materials and Methods

### 2.1. Patient Population

This retrospective study, based on analysis of electronic medical records, was reviewed and exempted by the Institutional Review Board of Chung Shan Medical University Hospital (IRB No. CS2-25036), in accordance with institutional regulations and the Declaration of Helsinki. The study included patients who underwent TAE between March 2020 and October 2024 for CPSP secondary to prior musculoskeletal surgery, trauma, or invasive procedures. The inclusion criteria for TAE were: (1) persistent moderate-to-severe pain or discomfort (Numeric Rating Scale [NRS] ≥ 5) lasting for more than three months, (2) failure to achieve adequate relief despite at least one month of conservative treatment (physical therapy and oral medications), and (3) inadequate response to at least one invasive therapy. Exclusion criteria included local infection, advanced atherosclerosis at the planned arterial access site, or a bleeding tendency.

Six patients met the inclusion criteria (Table 1). Pre-TAE MRI in all patients demonstrated increased high T2 signal intensity and/or increased contrast enhancement in the corresponding painful region, indicative of persistent inflammation and angiogenesis. Prior to undergoing TAE, all patients received a detailed explanation of the available management strategies, including potential risks and benefits, and provided written informed consent for the procedure. All clinical data were retrospectively collected by the author (N.-Y.C.) from the electronic medical record system of Chung Shan Medical University Hospital. Data extraction was conducted using a predefined chart review protocol developed by the study team to ensure consistency. The hospital operates a health information system, and all clinical parameters, procedural details, and follow-up outcomes were extracted directly from this digital system. No paper-based records were used in this study. To ensure data accuracy and completeness, the abstraction process was independently verified by a second investigator (K.-W.L.).

### 2.2. TAE Procedure

All TAE procedures were performed by a single experienced interventional radiologist (K.W.L.). Patients were treated based on the physician’s clinical judgment using either a traditional TAE approach with a 5-F sheath, catheter, and microcatheter or a simplified TAE technique involving direct arterial puncture and infusion of imipenem/cilastatin (IPM/CS) particles into the nearest artery. For traditional TAE, digital subtraction angiography was performed in the painful region using a 4-F diagnostic catheter (JR4, Terumo, Tokyo, Japan) with the injection of 15 mL of iodinated contrast medium (Xenetix 350^®^, Guerbet, Villepinte, France) at a rate of 3 mL/s. Superselective catheterization of suspected branches was achieved using either a 4-F or 5-F catheter (JR4, Terumo, Tokyo, Japan, or RIM, Cook Medical, Bloomington, IN, USA), followed by a 1.98-F microcatheter (Masters Parkway Soft, Asahi Intecc, Aichi, Japan). Superselective angiography was performed with a slow, manual injection of 1–2 mL of contrast agent. If abnormal staining was observed or the patient reported evoked pain during selective angiography, defined as reproducible pain or a heat sensation corresponding to their usual symptoms, embolization was initiated.

Traditional TAE was performed using a suspension of IPM/CS, 500 mg in 10 mL of iodinated contrast, delivered in 0.5–1 mL increments until transient stasis of antegrade blood flow was achieved for 3–5 heartbeats in the embolized vessel. Typically, 1–2 mL of the IPM/CS mixture was required to reach the treatment endpoint in each branch. Following the procedure, the catheter and introducer sheath were removed, and hemostasis was achieved using either manual compression or a vascular closure device (TR Band, Terumo, Tokyo, Japan, for radial artery access, or StarClose, Abbott, Abbott Park, IL, USA, for femoral artery access).

One patient underwent a simplified TAE procedure, in which 2 mL of IPM/CS was infused via radial artery access to treat persistent pain at the base of the finger following injection therapy for stenosing tenosynovitis. To prevent nontarget embolization of the digital arteries, a sterile rubber glove was tightly wrapped around the base of the finger to function as a manual tourniquet. Compression was applied for approximately 5 min during IPM/CS infusion and was adjusted to remain just below the patient’s tolerance level to avoid digital ischemia, as confirmed by the maintenance of capillary refill distal to the band. The tourniquet was removed approximately one minute after the infusion was completed. Hemostasis at the puncture site was achieved via manual compression.

Patients were monitored for one to two hours postprocedure before discharge. If residual or recurrent pain was reported during follow-up, an additional TAE session was permitted at the clinician’s discretion at least four weeks after the initial procedure, with the treatment date documented in the medical records. Patients were allowed to continue their pre-existing conservative treatment regimens and initiate previously intolerable therapies once pain had sufficiently improved, with rationale documented for each case. To minimize confounding effects, no new therapies were initiated between baseline and 2-week follow-up, allowing clearer attribution of early pain reduction to TAE alone.

### 2.3. Post-TAE Evaluation

Technical success, adverse events, and changes in NRS scores were assessed. Technical success was defined as the successful intra-arterial delivery of IPM/CS with achievement of the intended embolization endpoint. Pain was evaluated using the NRS at baseline, between 2–4 weeks, between 4–8 weeks, and at the last follow-up, which occurred beyond 12 weeks postembolization. Clinical success was defined as a reduction of at least 50% in the NRS pain score at the last follow-up compared with baseline. Adverse events were documented and classified according to the guidelines established by the Society of Interventional Radiology [12].

### 2.4. Statistical Analysis

Baseline and follow-up NRS scores were analyzed using a linear mixed model (LMM) to assess changes in NRS scores before treatment and at each follow-up time point after TAE. Pairwise comparisons were performed using estimated marginal means with Bonferroni adjustment, using baseline as the reference. Technical and clinical success rates were reported as percentages. All statistical analyses were performed using SPSS, version 25 (IBM, Armonk, NY, USA). A two-sided *p*-value < 0.05 was considered statistically significant for all tests.

## 3. Results

All six included patients had previously received various nonsurgical and/or surgical interventions, with symptoms persisting for more than three months. The mean duration of symptoms prior to undergoing TAE was 20.5 ± 17.4 months (range: 5–48 months). One patient was self-referred, while the remaining five were referred to our interventional radiology outpatient clinic by orthopedic surgeons, physical medicine and rehabilitation specialists, or interventional pain physicians. The mean age of the cohort was 46.9 ± 17.4 years. Clinical presentations included pain at the base of the finger (*n* = 2), ankle pain with stiffness (*n* = 3), and anterior knee pain (*n* = 1). Preprocedural MRI demonstrated findings such as periligamentous edema, arthrofibrosis, and increased contrast enhancement in areas corresponding to the reported pain, features indicative of pathological neovascularization. The patient cohort’s baseline demographic and clinical characteristics are summarized in Table 1.

Among the six patients, five underwent a second TAE procedure because of partial pain relief following the initial treatment. The mean interval between the first and second TAE sessions was 58.8 ± 33.9 days. A total of 11 TAE procedures were performed, during which abnormal angiogenesis was consistently identified, and the procedural endpoint was achieved in all cases, yielding a technical success rate of 100%. The mean dose of IPM/CS per procedure was 352.3 ± 175.5 mg.

Three patients presenting with ankle pain had MRI evidence of ankle arthrofibrosis. Prior to TAE, attempts at ultrasound-guided percutaneous fibrotic tissue release and prolotherapy/regenerative injection were unsuccessful because of severe intraprocedural pain. Following TAE, all three patients experienced partial pain relief and subsequently underwent successful ultrasound-guided fibrotic tissue release and injection therapy (dextrose or bone marrow aspirate), administered biweekly. The median follow-up duration after TAE was 13 months (interquartile range, 7.25–39.0 months). Pain relief was statistically significant over time. The mean NRS score decreased from 7.8 ± 1.2 at baseline to 4.3 ± 0.8 at 2–4 weeks, 2.5 ± 0.8 at 4–8 weeks, and 1.3 ± 1.2 at the final follow-up (≥3 months post-treatment). Linear mixed model analysis revealed a significant main effect of time on pain scores (*p* < 0.001). Pairwise comparisons using estimated marginal means with Bonferroni correction showed significant reductions in NRS scores from baseline to each follow-up point (baseline vs. 2–4 weeks: *p* = 0.001; baseline vs. 4–8 weeks: *p* < 0.001; baseline vs. final follow-up: *p* < 0.001). A post hoc power analysis using G*Power (version 3.1.9.7) was performed based on the observed reduction in NRS from baseline (mean = 7.8, SD = 1.1) to the final follow-up (mean = 1.3, SD = 1.2). The estimated effect size (Cohen’s f ≈ 2.0) yielded a statistical power greater than 0.99 for a repeated-measures design with four time points and six participants, confirming sufficient power to detect treatment-related changes. These findings suggest a consistent and progressive improvement in pain following TAE. All patients achieved at least a 50% reduction in pain at final follow-up compared with baseline, indicating a clinical success rate of 100%.

Follow-up MRI was available for three patients at 3, 6, and 8 months post-TAE, respectively, and all demonstrated significant resolution of inflammation. Notably, two patients with ankle arthrofibrosis, who previously had severely impaired mobility and were dependent on assistive devices (axillary crutches and wheelchair), were able to ambulate independently following sequential TAE and adjunctive therapies.

No major adverse events occurred. One patient experienced moderate subcutaneous hemorrhage at the femoral artery access site, which resolved spontaneously within one week. There were no cases of tissue necrosis, dermal ulceration, tendon rupture, or peripheral paresthesia in any embolized region. Intraprocedural and postprocedural details are summarized in Table 2 and further described in the following section.

## 4. Detailed Case Description

### 4.1. Persistent Finger Base Pain Postsurgical Tenolysis and Percutaneous Injection

**Figure 1:**
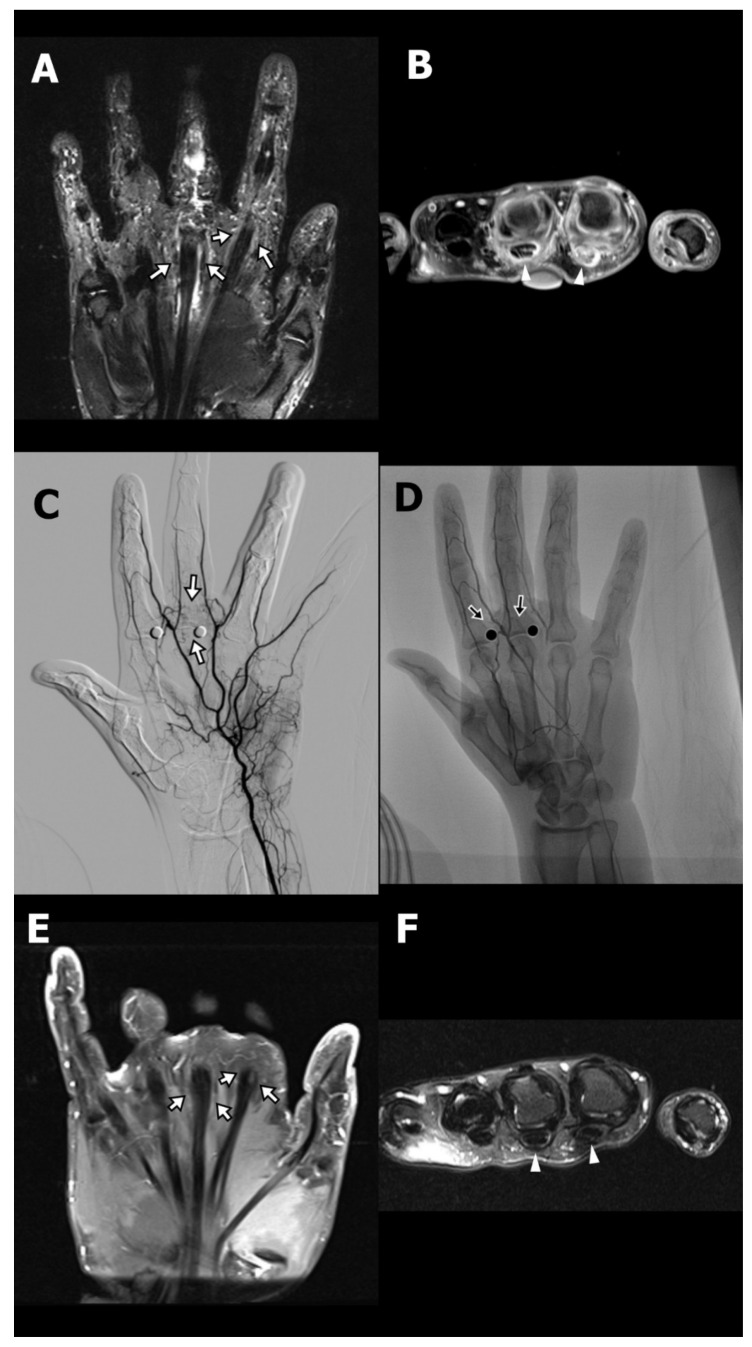
Preprocedural coronal (**A**) and axial (**B**) T2-weighted fat-suppressed magnetic resonance images (MRI) of a 36-year-old female presenting with persistent pain and swelling at the base of the fingers, two years after undergoing tenolysis. The images demonstrate thickening of the flexor tendon sheaths and tendons, along with increased peritendinous T2 hyperintense fluid accumulation around the middle and index fingers at the A1 pulley level (arrows in (**A**); arrowheads in (**B**)). Selective digital subtraction angiography of the left palmar arch revealed abnormal angiogenesis arising from palmar digital arterial branches (arrows) corresponding to the painful region (**C**). Transarterial embolization was performed using imipenem/cilastatin particles in the targeted arterial branches (arrows) (**D**). Follow-up MRI at 6 months postembolization demonstrated decreased inflammatory changes and reduced peritendinous fluid on post-gadolinium-enhancement T1-weighted fat-suppressed imaging (**E**) and T2-weighted fat-suppressed imaging (**F**) (arrows in (**E**); arrowheads in (**F**)).

A 36-year-old female (Case 1) with a medical history of type 2 diabetes mellitus, managed with oral hypoglycemic agents, presented with chronic pain at the base of the left index and middle fingers, accompanied by restricted flexion. She had been diagnosed with stenosing tenosynovitis two years prior. Initial treatment included A1 pulley tenolysis, which paradoxically resulted in exacerbation of pain and swelling at the finger base, exceeding preoperative levels. Subsequently, she underwent revision surgery and received three local corticosteroid injections. Despite these interventions, her symptoms persisted, prompting referral to the interventional radiology clinic for evaluation of TAE as a therapeutic option. On physical examination, the patient exhibited marked hyperalgesia at the base of the affected fingers, with disproportionate pain elicited by light palpation. Although full flexion of the fingers was achievable, it was associated with discomfort due to significant swelling. MRI revealed pronounced peritendinous and intratendinous edema with fluid accumulation at the A1 pulley region of the index and middle fingers (Figure 1A,B).

TAE was performed via a transfemoral arterial approach. Angiography identified abnormal angiogenesis arising from the palmar digital arterial branches supplying the symptomatic region (Figure 1C). Superselective catheterization of the affected arterial branches was achieved using a microcatheter, and embolization was performed using IPM/CS particles, delivered in the target vasculature (Figure 1D; arrows). Following the initial TAE procedure, the patient’s NRS decreased from 9 to 3 at two weeks, with sustained improvement at five weeks. A second TAE session was conducted 77 days after the initial intervention, resulting in complete pain resolution (NRS 0) at the six-month follow-up, which remained stable for over two years. Follow-up MRI at six months post-TAE demonstrated complete resolution of peritendinous fluid and improvement in both peritendinous and intratendinous edema (Figure 1E,F).

Another companion case involved a 56-year-old female (Case 2) who presented with chronic right middle finger pain and restricted flexion following a series of corticosteroid injections for stenosing tenosynovitis. Although the patient had not undergone surgery, she developed progressively worsening pain characterized by hyperalgesia, swelling, and functional limitation—symptoms consistent with CPSP, as defined by the International Association for the Study of Pain (IASP) and the International Classification of Diseases, 11th Revision. MRI demonstrated peritendinous inflammation at the A1 and A3 pulley levels (Supplementary Figure S1A). Standard conservative treatments, including physical therapy, nonsteroidal anti-inflammatory drugs (NSAIDs), and weak opioids, were ineffective. Given the persistent symptoms and imaging evidence of localized pathological angiogenesis, a simplified TAE procedure was pursued. Flow restriction of nontarget digital arteries was achieved with a sterile rubber glove tourniquet (Supplementary Figure S1B). Digital subtraction angiography revealed hypervascularity in the symptomatic region (Figure 1C), and embolization was performed using IPM/CS particles (Figure 1D). The patient’s Numeric Rating Scale (NRS) pain score decreased from 8 at baseline to 5 at two weeks, and 3 at four weeks, accompanied by improved finger function and reduced reliance on analgesics. A second TAE session resulted in complete resolution of pain (NRS 0) at both three- and six-month follow-ups.

### 4.2. Ankle Arthrofibrosis Postoperative Case

**Figure 2:**
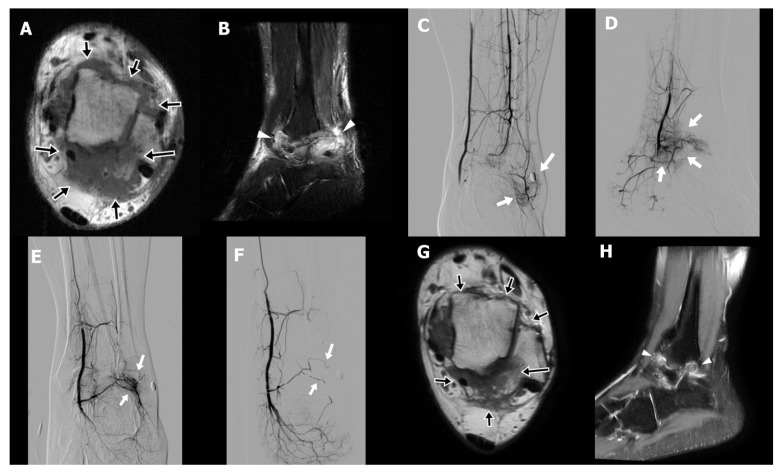
Preprocedural axial T1-weighted magnetic resonance image (MRI) (**A**) of a 31-year-old male with chronic right ankle pain persisting for 4 years following fracture fixation demonstrates dense fibrotic tissue with intermediate signal intensity along the capsule of the tibiotalar joint, predominantly in the posterolateral aspect (arrows in (**A**)). The sagittal T2-weighted fat-suppressed image (**B**) reveals heterogeneous high signal intensity consistent with a diffuse inflammatory process (arrowheads in (**B**)). Selective digital subtraction angiography (DSA) of the peroneal artery (anterior–posterior projection (**C**)), posterior tibial artery (lateral projection (**D**)), and anterior tibial artery (anterior–posterior projection, (**E**)) demonstrates abnormal angiogenesis (arrows in (**C**–**E**)) corresponding to the patient’s area of maximal pain, which is marked externally by a circular skin marker. Post-embolization angiography of the anterior tibial artery (**F**) shows resolution of the abnormal vascular blush (arrow in (**F**)) while preserving normal arterial branches. Follow-up MRI at 6 months reveals decreased fibrotic tissue in the posterolateral tibiotalar joint capsule on axial T1-weighted imaging (arrows in (**G**)), and reduced T2 hyperintensity on sagittal imaging (arrowheads in (**H**)), indicating regression of inflammation compared with baseline.

A 31-year-old male technician (Case 3) presented with chronic right ankle pain and limited plantar flexion persisting for four years following open reduction and internal fixation of an ankle fracture. Despite comprehensive conservative management, including physical therapy, oral NSAIDs, implant removal after fracture healing, and extracorporeal shock wave therapy, his symptoms remained refractory. On physical examination, the patient exhibited hyperalgesia and reported electric-shock-like sensations upon palpation of the posterolateral ankle, indicative of neuropathic pain. MRI demonstrated periarticular fibrotic tissue with increased T2 signal surrounding the tibiotalar joint, particularly in the posterolateral aspect, consistent with arthrofibrosis (Figure 2A,B). An initial attempt at percutaneous needle release of fibrotic tissue combined with prolotherapy was unsuccessful because of intolerable procedural pain. Subsequent diagnostic angiography revealed abnormal angiogenesis involving all three major arterial branches of the lower leg (Figure 2C–E). TAE was performed until complete occlusion of the pathological neovasculature was achieved (Figure 2F). The patient reported early pain relief, with the NRS decreasing from 8 to 5 by the second day following TAE. This improvement allowed for successful and tolerable completion of percutaneous fibrotic tissue needle release in conjunction with dextrose and bone marrow aspirate injections. A second TAE was conducted three months after the initial procedure. Six weeks postintervention, the patient’s NRS further declined to 3, with sustained pain relief observed at both six- and nine-month follow-ups. Follow-up MRI at six months demonstrated a significant decrease in both arthrofibrotic tissue and associated soft tissue edema (Figure 2G,H).

The two additional cases of ankle arthrofibrosis, following pes planus correction (Case 4) and fracture fixation (Case 5) respectively, are presented in Supplementary Figures S2 and S3.

### 4.3. Anterior Knee Pain Following Patella Fracture Surgery

A 63-year-old male patient (Case 6) presented with persistent anterior knee pain following open reduction and internal fixation for a patellar fracture. His symptoms included localized swelling, restricted range of motion, and severe pain exacerbated by squatting or kneeling. Despite undergoing implant removal, followed by three months of physical therapy and oral NSAIDs, his symptoms remained refractory to conventional management.

**Figure 3:**
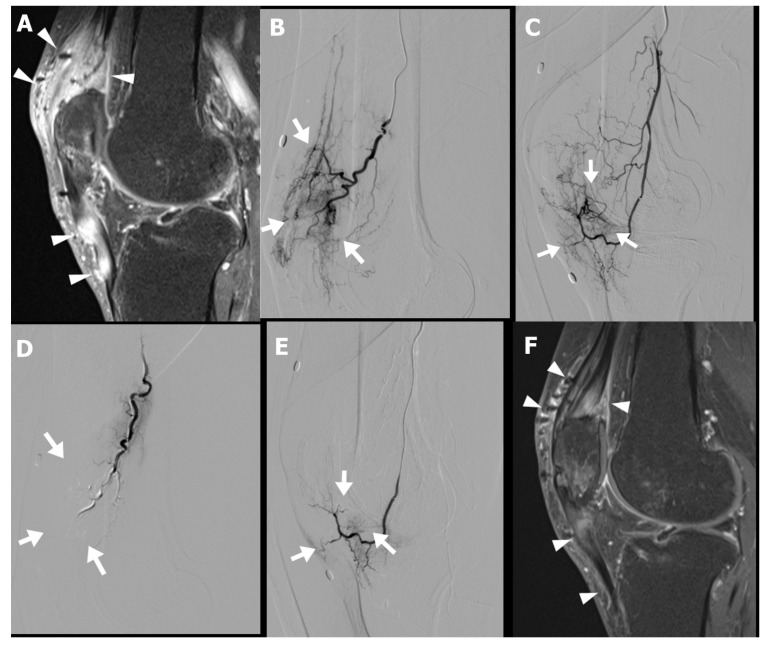
Preprocedural gadolinium-enhanced sagittal T1-weighted fat-suppressed magnetic resonance image (MRI) (**A**) of a 63-year-old male with chronic anterior knee pain persisting for 8 months following patellar fracture fixation demonstrates diffuse peritendinous and intratendinous contrast enhancement involving both the quadriceps and patellar tendons, as well as the adjacent soft tissue (arrowheads). Selective digital subtraction angiography of the superior patellar artery (lateral projection, (**B**)) and descending genicular artery (lateral projection, (**C**)) reveals abnormal angiogenesis (arrows in (**B**,**C**)) corresponding to the area of inflammation noted on MRI. Postembolization angiography (**D**,**E**) shows resolution of the abnormal vascular blush (arrows in (**D**,**E**)) indicating successful embolization of the pathological neovasculature. Follow-up MRI at 3 months postembolization demonstrates reduced peritendinous and intratendinous contrast enhancement (arrowheads in (**F**)), indicating decreased inflammation compared with the pretreatment MRI.

Musculoskeletal sonography and MRI demonstrated increased peritendinous edema and hyperemia surrounding the quadriceps and patellar tendons (Figure 3A). Diagnostic catheter-based angiography revealed pronounced hypervascularity in the superior patellar artery and descending genicular artery territory (Figure 3B–E). The patient subsequently underwent TAE targeting these vascular territories in two sessions.

Following the procedure, the patient reported gradual improvement in anterior knee pain, with the NRS decreasing from 6 at baseline to 4 at three weeks, and further to 2 at the three-month follow-up. Follow-up MRI demonstrated a marked reduction in both peritendinous and intratendinous angiogenesis (Figure 3F). Clinically, the patient regained full functional capacity, with resolution of swelling and the ability to kneel and squat without discomfort.

## 5. Discussion

CPSP is often an underrecognized clinical condition, characterized by persistent discomfort that is disproportionate to the expected postoperative healing trajectory and not attributable to unsuccessful treatment or incomplete resolution of the original pathology. It typically presents with neuropathic pain characteristics, including sensations described as burning, stabbing, or electric-shock-like, and is commonly associated with hyperalgesia or allodynia due to central and/or peripheral sensitization. It is critical to differentiate CPSP from inadequate treatment or unresolved pathology related to the original surgical condition, as the therapeutic strategies differ significantly.

The underlying mechanisms of CPSP are complex, predominantly neuropathic but potentially also including nociceptive and inflammatory components [3]. Additionally, abnormal angiogenesis could occur within the affected tissues, as demonstrated in our presented cases. In our experience, TAE achieved technical success in all six CPSP patients, demonstrating a 100% clinical success rate. Significant pain reduction was achieved through the targeted elimination of pathological angiogenesis, and importantly, no major adverse events were reported.

Adhesive capsulitis, an inflammatory condition in the shoulder with unknown etiology, has been successfully treated with TAE in both human and animal models [7,13]. Its anti-inflammatory efficacy has been validated through comparative pathological assessments, MRI, and [18F]-fluoro-2-deoxyglucose positron-emission tomography/computed tomography [7,8,14]. Similarly, CPSP, which involves chronic inflammatory processes coupled with abnormal angiogenesis, has shown significant clinical improvement and resolution of inflammation on MRI following TAE. These findings further reinforce the potential application of TAE in managing inflammatory diseases of the musculoskeletal system.

For pain physicians, performing interventional procedures in CPSP patients can be challenging, as hyperalgesia or allodynia may render direct percutaneous interventions intolerable, as seen in our cases 2–5. Alternative therapeutic options, such as regional nerve blocks, nerve ablation, or spinal cord stimulation, may alleviate pain but fail to address localized inflammatory processes directly. TAE, performed through distal arterial access, offers a targeted approach to managing inflammation without direct contact with already sensitized skin/tissue. This method could significantly alter current therapeutic strategies. In our cases of postsurgical arthrofibrosis, all patients experienced substantial pain reduction following TAE, which subsequently facilitated further percutaneous treatments. Thus, incorporating TAE into multifaceted CPSP treatment regimens has considerable potential to improve patient outcomes. In our cohort, all patients had undergone multiple prior interventions without satisfactory results. Notably, percutaneous treatments such as fibrosis needle release and prolotherapy were attempted but aborted because of intolerable pain. These same interventions were successfully completed only after TAE-induced pain relief, supporting the interpretation that TAE was the primary driver enabling subsequent functional improvement, rather than a confounding therapeutic variable.

Our study has several limitations. First, the small sample size and retrospective design may introduce selection bias and limit the generalizability of our findings. Second, inconsistencies in follow-up duration and methodology, influenced by variable referral practices, highlight the need for standardized, long-term monitoring to accurately assess outcomes and recurrence. Furthermore, although functional improvement was observed clinically, objective functional assessments were not systematically performed because of variability in patient follow-up. In addition, the cost of TAE may represent a significant barrier to broader clinical adoption. The procedure requires dedicated infrastructure and skilled personnel, including an angiosuite, trained interventional radiologists and radiographers, and single-use materials such as catheters, microcatheters, and guidewires. These factors collectively contribute to the procedural cost. While no formal cost analysis was performed in this study, future research should assess the cost-effectiveness and health-economic implications of TAE to inform broader clinical implementation and health policy decision-making.

Despite these limitations, our findings suggest that TAE may provide effective pain relief and broaden therapeutic options for CPSP patients. Future studies with larger sample sizes, prospective designs, standardized assessments, and longer follow-up durations are warranted to confirm the efficacy and durability of TAE. Comparative trials between TAE and conventional treatments, incorporating standardized outcome measures and patient-reported metrics, would help clarify the specific benefits of embolization. Such studies would strengthen causal inference and support evidence-based clinical decision-making.

## 6. Conclusions

TAE may serve as a feasible and safe therapeutic option for patients with CPSP associated with pathological angiogenesis, particularly in cases refractory to standard treatments. In this preliminary experience, TAE was associated with notable pain reduction, absence of major adverse events, and improved tolerance for subsequent therapies. These findings suggest a potential role for TAE in the multidisciplinary management of CPSP, especially when conventional interventions are limited by pain sensitivity or procedural intolerance. However, given the small sample size and retrospective design, further prospective studies with larger patient populations and longer follow-up are necessary to validate these observations and better define the long-term efficacy of TAE in this context.

## Figures and Tables

**Table 1:** Demographic and clinical characteristics of included patients.

Case No.	Gender/Age (Years)/BMI (kg/m^2^)	Pain Location	Prior Disease/Event	Symptom Duration (Months)	Referral Source	Treatments Prior to TAE
1	Female/36.1/33.0	Finger base	Stenosing tenosynovitis/surgical tenolysis	14	Orthopedic surgeon	Oral NSAIDs and weak opioid, corticosteroid injection
2	Female/55.7/25.0	Finger base	Stenosing tenosynovitis/corticosteroid injection	12	PM&R physician	PT, oral NSAIDs and weak opioid
3	Male/30.7/32.8	Posterior ankle	Ankle fracture/ORIF	48	PM&R physician	Implant removal, PT, oral NSAIDs, ESWT, prolotherapy (attempted)
4	Male/27.8/22.1	Medial ankle	Pes planus/subtalar arthroereisis	5	PM&R physician	PT, oral NSAIDs, prolotherapy (attempted)
5	Female/67.9/19.7	Lateral ankle	Ankle fracture/ORIF	36	self-referred	Implant removal, prolotherapy, talocalcaneal fusion, prolotherapy (attempted)
6	Male/62.9/22.5	Anterior knee	Patella fracture, ORIF	8	Pain specialist	Implant removal, PT, oral NSAIDs
Mean ± SD	—/46.9 ± 17.4/25.9 ± 5.7	—	Median (IQR)	13 (7.25–39.0)	—	—

Abbreviations: BMI, body mass index; ESWT, extracorporeal shock wave therapy; IQR, interquartile range; NSAIDs, nonsteroidal anti-inflammatory drugs; ORIF, open reduction and internal fixation; PM&R, physical medicine and rehabilitation; PT, physical therapy; SD, standard deviation; TAE, transarterial embolization.

**Table 2:** Summary of post-TAE outcomes.

Case No.	Preprocedure MRI Findings	TAE Procedures Received	NRS Baseline	NRS 2–4 Weeks	NRS 4–8 Weeks	NRS at Last Follow-Up (Time Point)/Functional Changes
1	Peritendinous inflammation	2	9	3	3	0 (24 months)/Finger flexion without pain
2	Peritendinous inflammation	2	8	5	3	0 (6 months)/Finger flexion without pain
3	Arthrofibrosis	2	8	5	3	3 (9 months)/Needle injection tolerable, ROM improved
4	Arthrofibrosis	1	9	5	1	1 (9 months)/Needle injection tolerable, crutch-free
5	Arthrofibrosis	2	7	4	3	2 (13 months)/Needle injection tolerable, wheelchair-free
6	Peritendinous inflammation	2	6	4	2	2 (3 months)/Able to kneel and squat
Mean ± SD	-	-	7.8 ± 1.1	4.3 ± 0.8 *	2.5 ± 0.8 **	1.3 ± 1.2 **/—

Abbreviations: MRI, magnetic resonance imaging; NRS, numeric rating scale; ROM, range of motion; SD, standard deviation; TAE, transarterial embolization. Notes: NRS 2–4 Weeks: NRS pain score recorded between two and four weeks after the first TAE. NRS 4–8 Weeks: NRS pain score recorded between four and eight weeks after the first TAE. NRS at Last Follow-Up: NRS pain score recorded at each patient’s latest follow-up, at least three months post-TAE. *p*-values were calculated using a linear mixed model with Bonferroni-adjusted pairwise comparisons versus baseline. *: *p* = 0.001 vs. baseline; **: *p* < 0.001 vs. baseline.

## Data Availability

The raw data supporting the conclusions of this article will be made available by the authors on request.

## Supplementary Materials

The following supporting information can be downloaded at: https://www.mdpi.com/article/10.3390/life15081208/s1, Figure S1: Representative imaging of simplified transarterial embolization (TAE) for persistent finger pain; Figure S2: A 27-year-old male with severe medial ankle pain following subtalar arthroereisis surgery experienced persistent symptoms despite implant removal, oral analgesics, and failed prolotherapy due to intolerable intraprocedural pain; Figure S3: A companion case of ankle arthrofibrosis in a 68-year-old female who developed persistent lateral ankle pain following fracture fixation, despite implant removal, talocalcaneal fusion, and failed prolotherapy due to intolerable intraprocedural pain.
