# Supplementary material for: Transarterial Embolization for Chronic Postsurgical or Posttraumatic Pain of Musculoskeletal Origin: Clinical Outcomes and Imaging Correlates
Source: Life (Basel). 2025 Jul 29;15(8):1208. doi: 10.3390/life15081208 (PMC12387131; doi:10.3390/life15081208)
Supplement: Supplementary file 1 [file life-15-01208-s001.zip › life-3748430-supplementary.pdf]

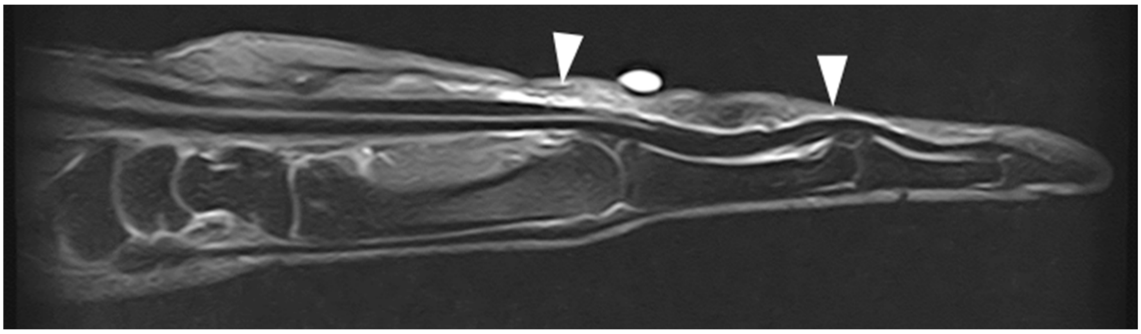

1A

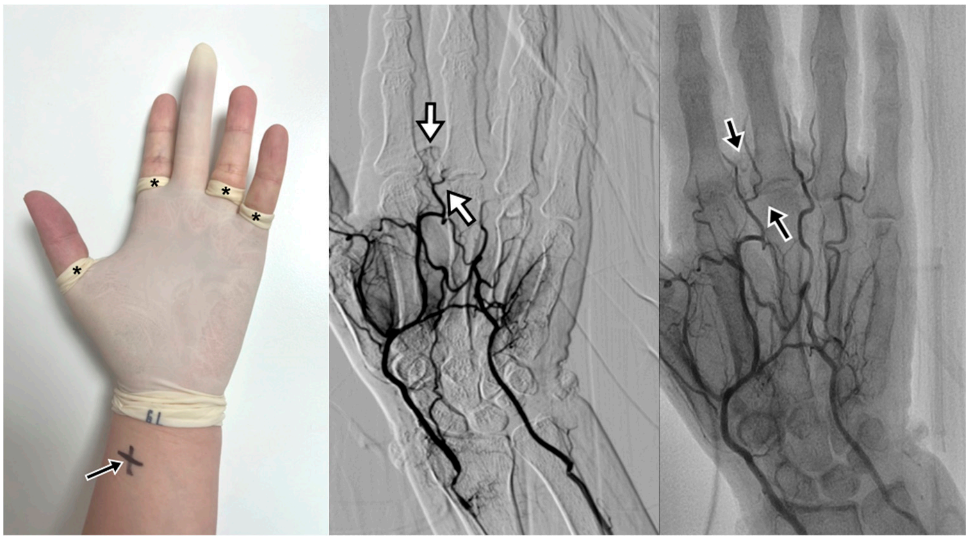

1B **1C** **1D**

Figure S1. Representative imaging of simplified transarterial embolization (TAE) for persistent finger pain. Sagittal T2-weighted fat-suppressed magnetic resonance image (A) shows peritendinous high signal intensity at the A1 and A3 pulley levels (arrowheads), consistent with active inflammation. A demonstration image (B) illustrates flow restriction using a sterile rubber glove as a manual tourniquet at the base of the non-involved fingers (asterisks), with the planned radial artery puncture site indicated by an ultrasound-guided skin mark (arrow). Digital subtraction angiography (C) confirms abnormal vascular blush at the base of the middle finger (arrows). Fluoroscopic imaging (D) demonstrates targeted delivery of imipenem/cilastatin particles into the pathological neovasculature (arrows).

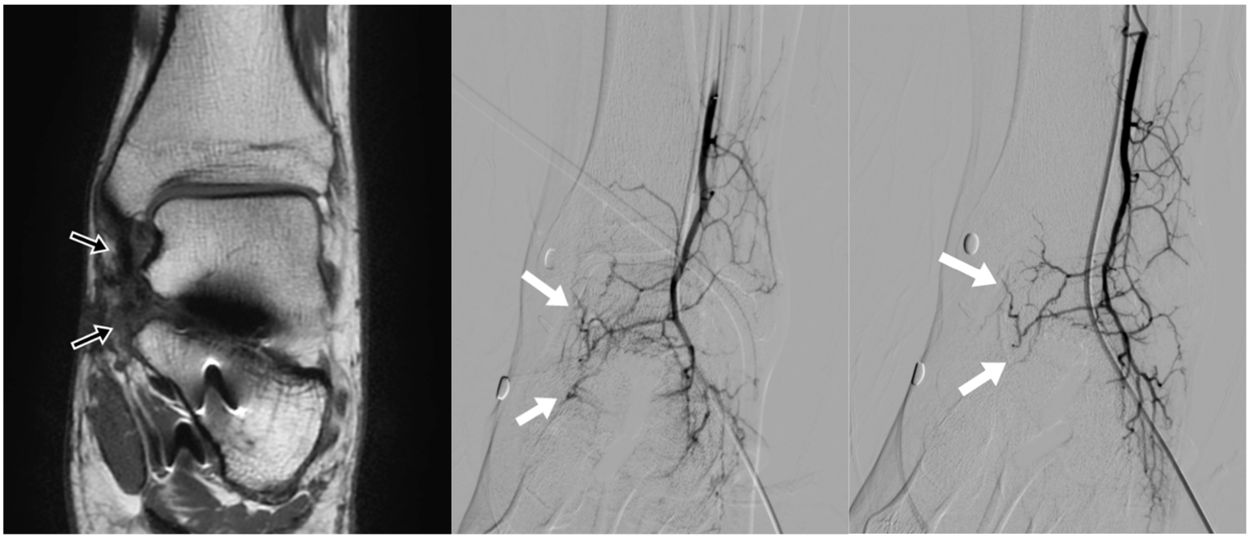

2A **2B** **2C**

Figure S2. A 27-year-old male with severe medial ankle pain following subtalar arthroereisis surgery experienced persistent symptoms despite implant removal, oral analgesics, and failed prolotherapy due to intolerable intraprocedural pain. Pre-embolization coronal T1-weighted MRI of the left ankle (2A) reveals intermediate signal intensity fibrotic tissue near the medial malleolus and talocalcaneal joint (arrows). Digital subtraction angiography (DSA) of the posterior tibial artery (2B) demonstrates abnormal angiogenesis in the region corresponding to the patient’s pain (arrows). Follow-up DSA post-embolization (2C) shows reduced abnormal vascular blush, indicating successful embolization. The patient's numeric rating scale (NRS) pain score improved from 9 to 5 at 2 weeks post-procedure, gradually decreased to 1 at 6 weeks, and remained at 1 at the 9-month follow-up following sequential needle scar release and physical therapy.

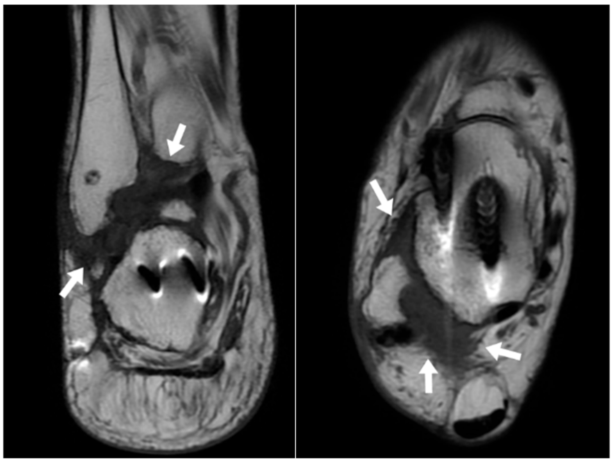

3A **3B**

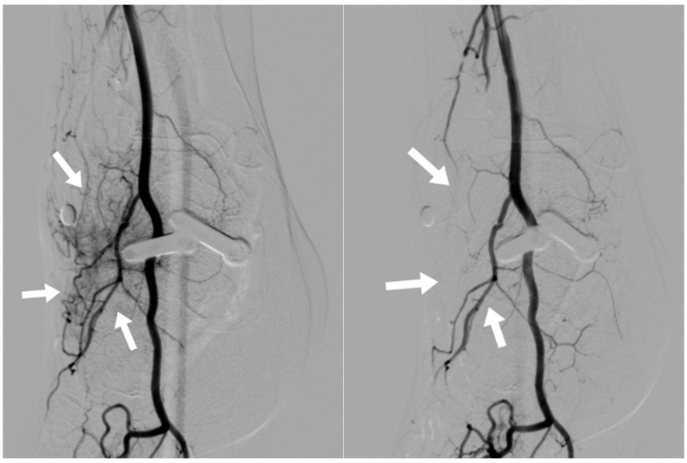

3C **3D**

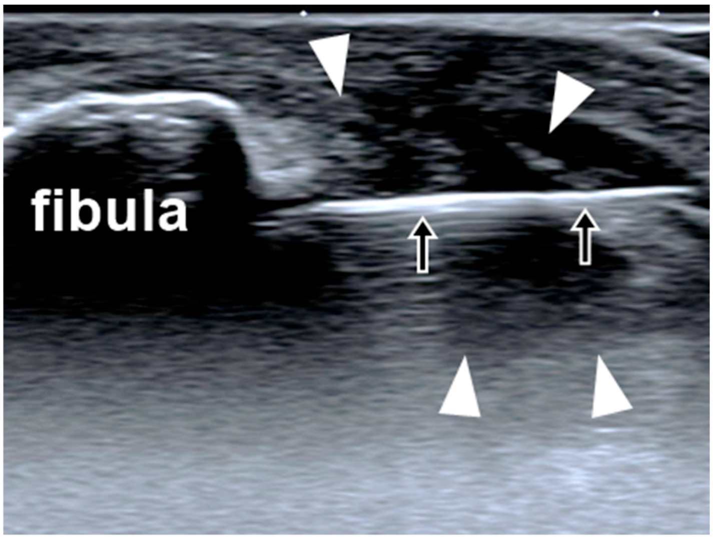

3E

Figure S3. A companion case of ankle arthrofibrosis in a 68-year-old female who developed persistent lateral ankle pain following fracture fixation, despite implant removal, talocalcaneal fusion, and failed prolotherapy due to intolerable intraprocedural pain. Coronal (3A) and axial (3B) T1-weighted magnetic resonance images demonstrate fibrotic tissue near the lateral malleolar region, showing signal intensity and morphology similar to that observed in Cases 3 and 4

(arrows). Digital subtraction angiography (DSA) of the anterior tibial artery (anterior–posterior projection, 3C) reveals abnormal angiogenesis arising from its branches (arrows). Follow-up DSA post-embolization (3D) shows resolution of the pathological vascular blush (arrows). Ultrasound-guided scar release using a 21-gauge needle along with bone marrow aspirate injection was performed multiple times following transarterial embolization. Ultrasound imaging (3E) demonstrates hypoechoic scar tissue (arrowheads) and the needle path (arrows). The patient's numeric rating scale (NRS) pain score improved from 7 to 4 at 2 weeks post-procedure, gradually decreased to 3 at 6 weeks, and 2 at 13 months. Initially wheelchair-dependent, she was able to ambulate independently by 3 months post-treatment.
